# Supplementary material for: MicroLib: A library of 3D microstructures generated from 2D micrographs using SliceGAN
Source: Sci Data. 2022 Oct 22;9:645. doi: 10.1038/s41597-022-01744-1 (PMC9588049; doi:10.1038/s41597-022-01744-1)
Supplement: Supplementary file 1 — Supplementary Information [file 41597_2022_1744_MOESM1_ESM.pdf]

Supplementary Information

A Microstructures of interest

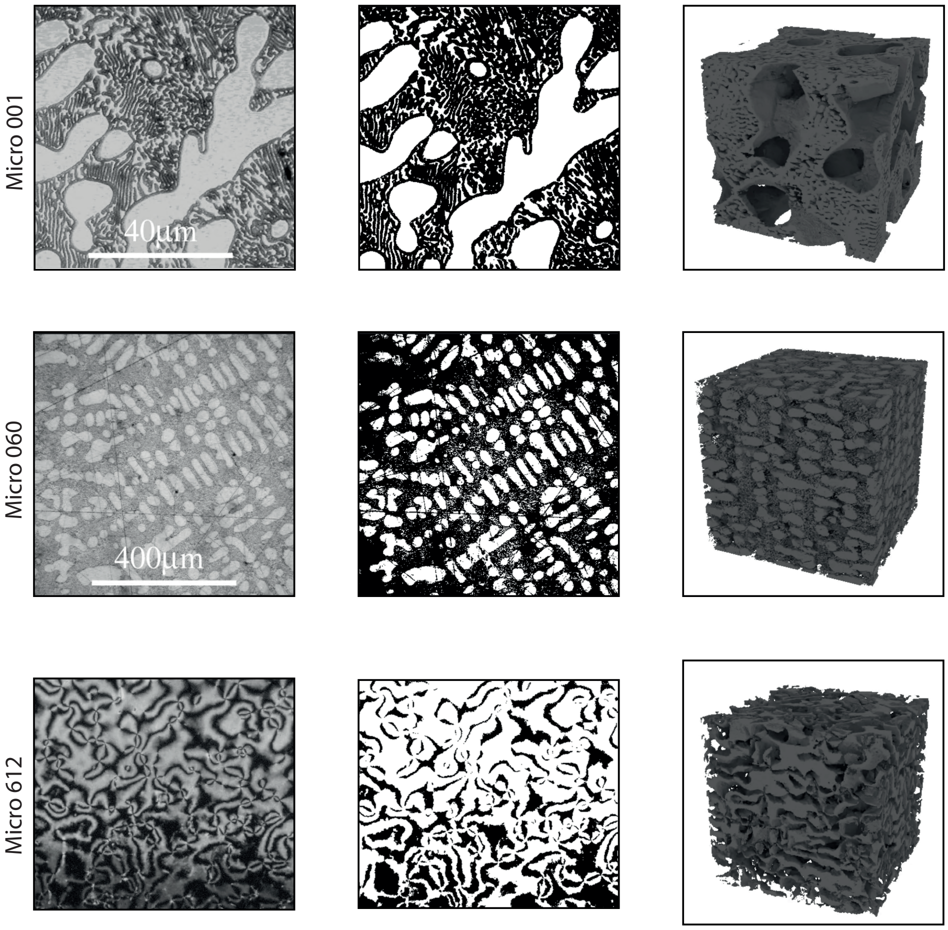

Figure S1: Examples showing potential modes of failure in *SliceGAN*. Micro 1 has large white features, which are not unlikely to be statistically representative of the microstructure as a whole. Micro 60 has a large number of ovals, which cannot be made into 3D features without introducing new features such as smaller circles. Micro 612 has a graded brightness, resulting in poor segmentation.
